# Scaling Up TB Screening and TB Preventive Treatment Globally: Key Actions and Healthcare Service Costs

**DOI:** 10.3390/tropicalmed8040214

**Published:** 2023-04-01

**Authors:** Srinath Satyanarayana, Carel Pretorius, Avinash Kanchar, Ines Garcia Baena, Saskia Den Boon, Cecily Miller, Matteo Zignol, Tereza Kasaeva, Dennis Falzon

**Affiliations:** 1 Centre for Operational Research, International Union against Tuberculosis and Lung Disease (The Union), New Delhi 110016, India; 2 Centre for Modelling and Analysis, Avenir Health, Glastonbury, CT 06033, USA; 3 Global TB Programme (GTB), World Health Organization, 1211 Geneva, Switzerland

**Keywords:** tuberculosis, prevention and control, latent tuberculosis infection, diagnosis, active case finding, costs and cost analysis

## Abstract

The 2018 United Nations High-Level Meeting on Tuberculosis (UNHLM) set targets for case detection and TB preventive treatment (TPT) by 2022. However, by the start of 2022, about 13.7 million TB patients still needed to be detected and treated, and 21.8 million household contacts needed to be given TPT globally. To inform future target setting, we examined how the 2018 UNHLM targets could have been achieved using WHO-recommended interventions for TB detection and TPT in 33 high-TB burden countries in the final year of the period covered by the UNHLM targets. We used OneHealth-TIME model outputs combined with the unit cost of interventions to derive the total costs of health services. Our model estimated that, in order to achieve UNHLM targets, >45 million people attending health facilities with symptoms would have needed to be evaluated for TB. An additional 23.1 million people with HIV, 19.4 million household TB contacts, and 303 million individuals from high-risk groups would have required systematic screening for TB. The estimated total costs amounted to ~USD 6.7 billion, of which ~15% was required for passive case finding, ~10% for screening people with HIV, ~4% for screening household contacts, ~65% for screening other risk groups, and ~6% for providing TPT to household contacts. Significant mobilization of additional domestic and international investments in TB healthcare services will be needed to reach such targets in the future.

## 1. Introduction

At the United Nations General Assembly High-Level Meeting (UNHLM) on tuberculosis (TB), convened on 26 September 2018, the heads of the UN Member States endorsed an ambitious political declaration to accelerate the progress towards the End TB Strategy targets [1]. The declaration included a commitment to detecting and treating at least 40 million people with TB between 2018 and 2022 (including 3.5 million children with TB and 1.5 million people with drug-resistant TB) and to providing TB preventive treatment (TPT) to at least 30 million high-risk individuals (6 million people with HIV, and 4 million children and 20 million adults who are household contacts of people with bacteriologically confirmed pulmonary TB).

Between 2018 and 2021, 26.3 million people with TB were reported to the WHO, equating to 66% of the target for TB case detection and treatment. During the same period, 12.5 million high-risk individuals were started on TPT (42% of the TPT target). The 2022 TPT target for people with HIV was exceeded between 2018 and 2021. In contrast, only 40% of the TPT target for household contacts aged <5 years and 3% of the target for contacts aged ≥5 years were achieved by 2021 [2]. This indicates major discrepancies in service delivery and an urgent need to strengthen and expand community outreach for TB detection, treatment, and TPT services.

Since early 2020, the coronavirus disease 2019 (COVID-19) pandemic has severely disrupted TB services across the globe [2]. In 2020, there was an 18% decline in the number of TB notifications to the WHO compared with 2019. TB notifications increased in 2021, but remained lower than pre-pandemic levels. The overall impact of the COVID-19 pandemic on the disruption of TB services has yet to be fully understood, but modeling indicates possible increases in TB incidence and mortality [3,4,5,6,7].

Systematic screening for TB disease and provision of TPT to people at risk are core components of the first pillar of WHO’s End TB Strategy, inherent to making the world TB-free by 2030 [8]. Achieving the UNHLM targets for TB detection and TPT was premised on enhancing TB diagnosis and treatment among those presenting at health facilities with TB symptoms (passive case finding) and systematic screening and TPT provision to the eligible high-risk groups [9]. Expanded use of chest radiography and better-performing diagnostic tools was envisaged as a more accurate and efficient strategy for TB case finding and TPT. New guidance on TB screening and TPT [10,11] has been released by the WHO since 2020 to inform implementers about the most appropriate, evidence-based approaches for implementation.

To inform future target-setting work, this paper describes the programmatic actions and direct healthcare service costs that would have been required to achieve the vision of the 2018 UNHLM regarding TB detection and TPT at the start of 2022, from a health system perspective, with adequate expansion of screening and TPT in various countries. In order to facilitate the presentation and discussions, we focused our estimates on the 33 countries with the highest burden of TB [12], which, altogether, comprised close to 90% of the global TB notifications in 2020 (Table 1).

## 2. Materials and Methods

### 2.1. Costing Approach

We followed the intervention costing method of the OneHealth Tool (OHT) [13] to model the estimated overall cost of TB detection and provision of TPT to household contacts of people with TB in 33 countries through the following steps: (a) the approximate number of people with TB to be detected and the number of household contacts to be initiated on TPT in order to reach the UNHLM targets were estimated; (b) the relevant WHO-recommended interventions and services were identified; (c) for each intervention, the size of the target population was identified, and for each service, the proportion of the target population in need of that service was determined; (d) the unit cost of each service in each country was determined; (e) unit costs were multiplied by the volume of services to obtain the total cost per intervention. The sum of the total costs of all interventions is expressed as a “price tag” for TB detection and TPT initiation to achieve the UNHLM targets in 33 countries. Each step is briefly explained below.

**Table 1:** Countries’ TB treatment targets and the estimated number of people remaining to be treated to achieve the UNHLM TB treatment targets in 30 high-TB burden countries and 3 countries on the global TB watchlist.

Country	Global Plan 2018–2022 TB Treatment Target (Number of Persons) [14]	Total Number of Persons Treated in 2018–2020 [2]	% Target Achieved 2018–2020	Estimated Shortfall of the Persons to be Treated
Angola	365,100	203,441	56%	161,659
Bangladesh	1,534,500	788,819	51%	745,681
Brazil	418,700	244,339	58%	174,361
Cambodia *	191,000	87,576	46%	103,424
Central African Republic	62,800	35,537	57%	27,263
China	3,857,400	2,148,225	56%	1,709,175
Congo	62,800	33,683	54%	29,117
Democratic People’s Republic of Korea	543,300	275,301	51%	267,999
Democratic Republic of the Congo	1,117,200	549,230	49%	567,970
Ethiopia	646,300	332,845	52%	313,455
Gabon	36,900	15,967	43%	20,933
India	11,900,000	5,700,307	48%	6,199,693
Indonesia	3,445,100	1,512,737	44%	1,932,363
Kenya	563,700	250,525	44%	313,175
Lesotho	40,500	18,735	46%	21,765
Liberia	52,500	23,054	44%	29,446
Mongolia	21,900	12,018	55%	9882
Mozambique	636,600	284,608	45%	351,992
Myanmar	734,600	375,846	51%	358,754
Namibia	42,900	22,118	52%	20,782
Nigeria	1,179,600	357,025	30%	822,575
Pakistan	2,225,300	961,774	43%	1,263,526
Papua New Guinea	151,600	86,785	57%	64,815
Philippines	2,450,000	1,037,376	42%	1,412,624
Russian Federation *	522,000	210,309	40%	311,691
Sierra Leone	97,700	50,644	52%	47,056
South Africa	1,023,800	628,618	61%	395,182
Thailand	472,400	258,655	55%	213,745
Uganda	321,500	182,619	57%	138,881
United Republic of Tanzania	561,100	240,691	43%	320,409
Viet Nam	535,000	302,013	56%	232,987
Zambia	205,700	111,221	54%	94,479
Zimbabwe *	111,900	61,940	55%	49,960
Grand Total	36,131,400	17,404,581	48%	18,726,819

* Countries on the Global TB Watchlist.

### 2.2. Estimating the Number of People with TB to Be Detected and the Household Contacts to Be Initiated on TPT to Achieve the UNHLM TB Detection and TPT Targets

From the Global Plan to End TB 2018–2022, we obtained the number of people with TB to be detected and the number of household contacts to be initiated on TPT between 2018 and 2022 for all 33 countries [14]. For each country, we then obtained the actual number of people with new and TB episodes and relapses and the number of household contacts initiated on TPT between 2018 and 2020 from the WHO Global Tuberculosis Report 2021 (Table 1 and Table 2) [2]. In addition, for the year 2021, we aggregated monthly or quarterly case detection data reported by the countries to the WHO [15]. Using this information, we derived the number of additional people with TB to be detected and the number of household contacts to be initiated on TPT which were necessary to achieve the UNHLM targets envisaged for 2022.

### 2.3. Identifying the Interventions and Services to Be Costed

We identified the interventions mentioned in Box 1 as necessary for achieving the UNHLM targets for TB detection and TPT initiation, focusing on interventions to increase the detection of people with bacteriologically confirmed pulmonary TB. To reach the target for household contacts to be initiated on TPT, it is essential to conduct contact investigation among people with TB identified through active TB case finding, in addition to contact investigation among people with TB who present to health services (passive case finding).

Box 1 **Interventions identified for costing.**

- Passive case finding focused on the detection of bacteriologically confirmed TB disease;
- Systematic screening of people living with HIV;
- Systematic screening of household contacts of all people with TB;
- Systematic screening for TB disease among high-risk groups, targeted towards those living in conditions with multiple risk factors for TB disease, such as in poor urban communities;
- TPT for all eligible household contacts of bacteriologically confirmed pulmonary TB disease detected through passive or active TB case finding.

For each intervention, the service components included in the costing model are summarized in Table 3. The algorithms depicting the services included under each intervention are given in the Supplementary Figures. The sensitivity and specificity of the screening algorithms were obtained from the WHO’s screening and diagnostic guidelines [10,16].

### 2.4. Estimating the Size of the Target Population for Interventions and Services

Based on the estimated numbers of TB patients still to be treated and people still to be given TPT in 2022 in order to reach the UNHLM targets (Table 1 and Table 2), we then calculated the number of people that would be need to be reached by different interventions using the assumptions in Table 4. We first estimated the number of TB patients likely to be detected through passive case finding, assuming diminishing disruption in TB detection due to COVID-19 post-2020. Detection increased in 2022 by 1.5 times the number of individuals detected with TB in 2020. We also assumed that the number of people with TB detected by passive case finding followed the same distribution of ages (<15 years, >15 years) and types of TB (new/retreatment, bacteriologically confirmed/clinically diagnosed, pulmonary/extrapulmonary) as reported by each country in 2020, and derived these estimates from OneHealth-TIME Estimates model outputs. We assumed that to detect one person with TB through passive case finding, roughly ten individuals with TB symptoms would require all diagnostic services listed under passive case finding.

The remainder of the TB patients would require detection by means of active case finding. For the different groups targeted by screening (i.e., people living with HIV, household contacts of all people with TB, and high-risk groups), we chose, from the available options described in the WHO operational handbook on systematic screening for TB disease, a screening and diagnostic algorithm that maximized both sensitivity and efficiency [17]. Generally, all those who screen positive will receive Xpert MTB/Rif^®^. To estimate the size of the target population for systematic screening of people living with HIV, we obtained the estimated number of persons who were newly diagnosed and on ART for less than 6 months, and the number of persons already diagnosed and on ART for more than 6 months, from the AIDS Impact Model in Spectrum [18]. We assumed that all persons newly diagnosed with HIV (and on ART for less than 6 months) would be screened for symptoms and, if symptomatic, receive a C-Reactive protein (CRP) blood test. We furthermore assumed that all persons living with HIV and on ART for more than 6 months would undergo symptom screening plus chest radiography at least once in the year 2022. The yield of systematic screening for TB disease among people living with HIV was calculated using data from the published literature [19].

To estimate the target population size for systematic screening of household contacts, we obtained data on the estimated household size for each country and the age structure of the country’s population, disaggregated by <5, 5–14, and ≥15 years [20,21]. The number of household members to be screened per index person with TB was calculated by subtracting one from the household size. We applied the age structure to the household members to obtain the number of the household contacts aged <5, 5–14, and ≥15 years who were eligible for systematic screening. All household contacts ≥15 years were assessed by symptom screening and chest radiography, while those <15 were screened for symptoms only. The yield of people with TB detected due to this systematic screening effort was calculated using data from the published literature [22].

To estimate the population size for systematic screening in high-risk groups, we estimated the number of people remaining to be detected after a scale-up of passive case finding and systematic screening of people living with HIV and household contacts. We then calculated that approximately 8% of the population with a relative risk of TB of at least 2.5 times that of the general population needed to be systematically screened for TB disease to cover this gap in case detection. These populations in informal urban settlements should be targeted by large-scale screening efforts [23,24,25]. In these populations, we assumed that those aged 18 years and above would undergo symptom screening and, if negative for symptoms, chest radiography.

For TPT, we assumed that at least 90% of the household contacts of people with bacteriologically positive TB would be eligible for a TB infection test (IGRA) and TPT after excluding those with TB disease (~3%) [22] and those with contraindications/refusals for TPT (~7%). In addition, we assumed that only 20% of eligible household contacts would undergo IGRA testing, and TPT would be initiated for all individuals with positive IGRA tests (~50%) [26,27]. Finally, we assumed that the remaining 80% of the household contacts would initiate TPT without a TB infection test.

### 2.5. Estimating Unit Cost for Each Service for Each Country

To estimate unit costs (in USD) for each service in the year 2022 for each of the 33 high-burden countries, we used the following methods:

We used the data from the Value TB project [28,29,30,31], which has recently been made available for five countries, namely, Ethiopia, Georgia, India, Kenya, and the Philippines. Four of these five countries (except Georgia) are among the thirty-three high-TB burden countries. For these four countries, we used local currency data for the base year and then inflate/convert methodology [32] to determine the unit costs for 2022.

For the remaining 29 countries, we reviewed the data on unit costs from country reports from the global health cost consortium [33] and used those values wherever available. When the unit cost data were not available, we extrapolated the unit costs from the five Value TB project countries. Georgia was used as a reference for the upper-middle-income high-TB burden countries (Brazil, China, Gabon, Namibia, Russian Federation, and Thailand). India was used as a reference for the lower-middle-income high-burden countries in South Asia (Bangladesh, Indonesia, Myanmar, and Pakistan). The Philippines was used as a reference for the middle-income high-burden countries in the Western Pacific region (Cambodia, Mongolia, Papua New Guinea, and Viet Nam). Kenya was used as a reference for the low-middle-income high burden countries in Africa (Angola, Congo, Lesotho, Nigeria, United Republic of Tanzania, Zambia, and Zimbabwe). Finally, Ethiopia was used as a reference for lower-income high-burden countries (Central African Republic, Democratic People’s Republic of Korea, Democratic Republic of the Congo, Liberia, Mozambique, Sierra Leone, and Uganda).

To extrapolate the unit cost from the reference country to the target country, we used an ingredients-based approach as suggested by Torres-Rueda S et al. [34]. Each cost input into the ingredients costing method was classified as tradable goods (consumables), non-tradable goods (overheads + capital costs), and staff costs. To convert the tradable goods from the reference country (R) to the target country (T), we converted the cost of the tradable goods into USD according to the base year. Then, using USD-based inflation rates, we adjusted the value to the target year, or we took the latest price, in USD, of the tradable goods as mentioned in the Stop TB Partnership Global Drug Facility’s drug or diagnostics catalogue [35,36]. For extrapolating costs of non-tradable goods (NT) from the base country to the target country, we used the ratio of purchasing power parity [37] to obtain the equivalent costs in local currency for the base year, inflated it in local currency using local country-specific inflation rates to the target year, and converted the result back to USD using the target year’s currency conversion rate. To convert staff costs (S) for a particular service from a base country to the target country, we used the staff time (in minutes) to conduct the activity and estimated the staff cost per minute in the base country. Staff time was extrapolated to the target country without any modifications from the base country. To convert the staff cost, we used the conversion rates from Serge et al. (2018) [38]. The GDP per capita multipliers presented in this paper, as well as the ratio of the nominal GDP per capita, were used to covert the staff wages per minute from the base country in the base year to the target country in the base year. We multiplied this cost by country-specific inflation rates, obtained the target yearly staff cost values in the local currency, and then converted those values to USD using the target year’s conversion rate. The total unit cost in the target country and target year is a sum of tradable goods, non-tradable goods, and staff time.

We obtained TB service delivery estimates from the Global TB Programme and used the WHO CHOICE health service delivery costs [39] of the most recent year to estimate the cost of outpatient visits. For the cost of drugs (for TPT) and consumables, we used the prices, in USD, from the latest Global Drug Facility diagnostic and medicine catalogs [35,36]. We assumed that CAD would cost an additional USD 1 per person (assuming high volumes) undergoing digital CXR. For all unit cost calculations, we used the latest country-specific GDP deflation and USD conversion rates published by the World Bank [40,41] to adjust the inflation and currency conversions from the base years to the target years (2021 and 2022).

### 2.6. Estimation of the Total Costs

The direct cost of a service for each type of intervention (si) in a particular country (c) is a product of the size of the target population for that service, the estimated proportion of the target population eligible for that service (vs), and the unit cost (uc):

i.e., direct cost_(si, c)_ = target population size_(si, c)_ × proportion of target population eligible for that service_(vs)_ × unit costs_(uc)_

The total cost for the interventions is the sum of all country values is given by $\sum_{c}^{\mathrm{si}}\mathrm{direct cost}\left(\mathrm{si},\;c\right)$.

## 3. Results

The volume of different services needed to reach the UNHLM targets derived from our costing model, aggregated across 33 HBCs and along with the average unit cost (weighted average across 33 countries), is shown in Table 5.

The number of people who would have needed to be initiated on TB treatment and the number of household contacts who would have needed to be initiated on TPT, disaggregated by the mode of detection (passive case finding, systematic screening of households, or systematic screening of high-risk groups), are reported in Table 6. Of the 12.6 million persons who needed to be notified from the 33 HBCs in order to reach the UNHLM targets, 9.7 million (77%) could have been detected by strengthening passive case finding, including among people living with HIV and attending health facilities for ART. In addition, about 780,000 individuals would have been assessed due to systematic screening of household contacts of people with TB, as discovered through passive case finding. The remaining 2.2 million individuals would have been detected by systematic screening of high-risk groups at the community level.

It is estimated that of the 12.6 million persons with TB who were initiated on TB treatment (Table 6), nearly 8 million (~65%) had bacteriologically confirmed TB (data not shown). About 90% of the household contacts of these 8 million persons, amounting to ~18.5 million household contacts in the 33 HBCs, would have been provided TPT.

Cost estimates for diagnosing 12.6 million people with TB and initiating 18.5 million household contacts on TPT are reported in Table 6. A total of USD 6.7 billion would have been needed for direct program costs in order to reach the UNHLM TB detection and TPT targets. The major cost driver (~USD 4.5 billion) was the systematic screening of high-risk groups at the community level. Passive case finding would have cost USD 991 million, USD 288 million would have been needed for the systematic screening of the household contacts of all people with pulmonary TB, and USD 648 million would have been required for the systematic screening of all people with HIV. To provide TPT to the household contacts of those with bacteriologically confirmed TB, USD 392 million would have been needed.

## 4. Discussion

We estimated that total direct healthcare system costs of at least USD 6.7 billion would have been necessary to achieve the UN’s TB case finding and TPT targets in 33 high-TB burden countries by the end of 2022. This amount exceeds the USD 5.4 billion which was available for all components of TB care in all low- and middle-income countries in 2021 [2]. Our cost estimate for TB prevention and screening alone aligns with the resource needs identified in the new Global Plan to End TB 2023–2030, which states that for this period, USD 209.8 billion will be needed for TB care and prevention. Excluding the costs of vaccination, this translates to an average of USD 19.65 billion per year for TB diagnosis, care, and prevention [42].

Our cost estimate is based on scaling up available technologies and currently feasible approaches. It is driven primarily by the need to undertake active TB case finding in high-risk groups at the community level. This includes the systematic screening of prisoners, miners, and populations with structural risk factors for TB, such as those living in poor urban communities and homeless communities, migrants, refugees, internally displaced persons, and other vulnerable or marginalized groups with limited access to healthcare [43]. Our model indicated that approximately 17% of people with TB had to be identified through high-risk group- and community-based screening to reach the UNHLM target. This proportion might be higher if case detection through passive case finding lags behind. In addition, our costing model assumes that at least ~8% of the population in high-TB burden countries with an estimated TB prevalence of >0.5% or TB incidence rate >2.5 times that of the general population will be reached by systematic screening. Implementing this is feasible and less costly in focused settings such as informal urban settlements [23]. The high costs of community screening of high-risk groups re due to the large volumes of people undergoing symptom screening and screening with CXR and CAD, as well as the substantial volume of people screening positive and having to receive the more expensive Xpert MTB/Rif testing.

Our data show that many people with TB can be detected through systematic screening of people living with HIV and their household contacts. Household contact investigation needs to be scaled up rapidly to cover the contacts of all people with TB. This will increase the number of TB cases detected and people started on TPT. The total cost of the provision of TPT to household contacts is low compared to the cost of case detection, even though we use 3HP (~USD 15 per person), which is more expensive than 6H, which is traditionally used for TPT (~USD 3 per person). Active case finding in people with HIV is essential, as they are at about an 18-times-higher risk of developing TB than the general population [2]. To minimize misdiagnosis or overdiagnosis of TB in people with HIV, it is important to use the screening tools that are recommended in the latest WHO guidelines on systematic screening [10]. This includes using newer, better screening tools, such as C-reactive protein and CXR, in addition to TB symptom screening [16].

In addition to expanding active case finding and screening, it is necessary to strengthen the diagnostic capacity to increase the detection of this disease among people presenting to health facilities with symptoms of TB by expanding access to mWRDs. mWRD testing has several advantages, including a short turnaround time, accurate results, and minimal technical training needed to run the test and interpret the results [44]. Additionally, these assays can identify rifampicin resistance, allowing for an early start with the appropriate treatment. For people without TB, this minimizes the chances of unnecessary treatment and isolation in hospitals or other institutions [10,16]. In some settings, molecular diagnostic platforms have been strengthened since 2020 as part of the COVID-19 pandemic response, and these could be leveraged for TB diagnostics.

The major limitations of our costing model include some of our assumptions. First, we assumed that it would have been possible to scale up mWRDs and CXR services within a very short period with 100% coverage. However, this may be less feasible in the near future, particularly in the context of the COVID-19 pandemic. Second, we did not include the costs of programmatic activities (e.g., training, advocacy, communication, social mobilization, supervision) to deliver people-centric and friendly TB services on the premise that these will be marked up with investments from existing resources. These activities and their related costs need to be identified and added to the direct program cost estimates provided in this study. Financial data submitted by various countries to the WHO indicate that the programmatic and patient support costs are highly variable, and range from 25% to 70% of the direct program costs [45]. Despite these limitations, our calculations have several strengths. Amongst others, our estimates consider the impact of COVID-19, and are, therefore, more relevant to current discussions. In addition, many of the utilized input values are derived from country sources elaborated upon over several years of reporting to WHO. We summed the totals of the estimates for individual countries instead of extrapolating modeled values throughout. The recommended interventions are based on the best available evidence and updated guidelines published by the WHO in 2020 and 2021. All proposed interventions are feasible, and are being implemented with available technologies worldwide.

## 5. Conclusions

The estimated direct health system costs to achieve the UNHLM targets for TB case detection and TPT in 33 high-TB burden countries alone exceeded the USD 5.4 billion available in 2021 for TB care in all 136 low- and middle-income countries who reported to the WHO. Our costing model indicates that a minimum of USD 6.7 billion would have been required for the direct health system costs of TB case detection and TPT in order to achieve the UNHLM targets in 33 high-TB burden countries, which comprise about 90% of TB incidence. With less than that amount reported available for all TB prevention, diagnosis, and treatment activities in 2021, and uncertainties regarding maintaining even these levels in the economic outlook post-2022, this is another eye-opener concerning the scale of additional funding needed to achieve some of the goals of the End TB Strategy. This analysis is, thus, useful when developing and assessing the costs of national health plans. The forthcoming UN High-Level Meeting in 2023 makes this more relevant. Future drives to achieve ambitious targets must be accompanied with adequate funding mobilization. This would entail increasing domestic funding and launching initiatives, such as the recent global drive that hugely expanded TPT coverage for people with HIV in several countries. Apart from state health agencies and donors, non-government organizations, employers, the private health sector, technical agencies, and community activists must be part of the post-pandemic recovery of TB services in order to increase health worker capacity and create demand. Only in this way can we aspire to enlarge the scale of screening, diagnosis, treatment, and preventive care of people affected by TB to the extent needed to bring the TB trends on track toward the End TB Strategy goals.

## Figures and Tables

**Table 2:** Countries’ TB preventive treatment (TPT) targets and the estimated number of household contacts to be provided TPT to achieve UNHLM TPT targets for 30 high-TB burden countries and 3 countries on the global TB watchlist.

Country	Global Plan 2018–2022 TPT Target (Household Contacts) [14]			Household Contacts Provided TPT in 2018–2020 [2]			Estimated Shortfall in the TPT Provision to Reach the UNHLM Targets
	Total	Age < 5 years	Age ≥ 5 years	Total	Age < 5 years	Age ≥ 5 years	
Angola	353,147	147,320	205,827	-	-	-	353,147
Bangladesh	962,256	300,630	661,626	79,688	77,697	1991	882,568
Brazil	216,655	57,980	158,675	25,991	3235	22,756	190,664
Cambodia *	63,113	18,680	44,433	16,537	5866	10,671	46,576
Central African Republic	45,745	17,995	27,750	1028	731	297	44,717
China	1,680,458	616,740	1,063,718	-	-	-	1,680,458
Congo	28,391	12,009	16,382	72	37	35	28,319
Democratic People’s Republic of Korea	256,137	101,220	154,917	33,541	31,373	2168	222,596
Democratic Republic of the Congo	1,055,585	465,660	589,925	101,685	101,685		953,900
Ethiopia	280,070	106,130	173,940	33,707	24,936	8771	246,363
Gabon	18,512	6945	11,567	-	-	-	18,512
India	5,944,997	1,720,060	4,224,937	283,738	283,738		5,661,259
Indonesia	1,589,642	438,330	1,151,312	18,140	17,705	435	1,571,502
Kenya	336,366	130,850	205,516	24,630	22,728	1902	311,736
Lesotho	20,817	7857	12,960	3796	2294	1502	17,021
Liberia	31,049	11,799	19,250	195	137	58	30,854
Mongolia	11,607	4214	7393	575	216	359	11,032
Mozambique	374,574	195,700	178,874	85,910	85,910	-	288,664
Myanmar	375,929	137,320	238,609	7264	3899	3365	368,665
Namibia	33,322	12,250	21,072	5248	4356	892	28,074
Nigeria	1,075,353	425,730	649,623	31,040	29,119	1921	1,044,313
Pakistan	1,602,246	514,000	1,088,246	16,152	15,980	172	1,586,094
Papua New Guinea	36,829	13,451	23,378	3806	2901	905	33,023
Philippines	915,803	266,540	649,263	14,287	9779	4508	901,516
Russian Federation *	238,650	88,940	149,710	20,609	20,609	-	218,041
Sierra Leone	92,930	35,550	57,380	-	-	-	92,930
South Africa	618,180	206,510	411,670	78,830	63,438	15,392	539,350
Thailand	265,272	97,070	168,202	22,763	11,492	11,271	242,509
Uganda	296,266	127,860	168,406	30,704	17,240	13,464	265,562
United Republic of Tanzania	296,759	119,990	176,769	25,775	25,231	544	270,984
Viet Nam	308,783	68,620	240,163	16,606	7464	9142	292,177
Zambia	118,211	50,680	67,531	10,080	6164	3916	108,131
Zimbabwe *	61,746	23,680	38,066	6388	6388	-	55,358
Grand Total	19,605,400	6,548,310	13,057,090	998,785	882,348	116,437	18,606,615

* Countries on global TB Watchlist.

**Table 3:** Screening and prevention interventions and associated service components to estimate the funding required to achieve the UNHLM case detection and TPT targets.

Interventions	Service Components
Passive case finding (among adults, children, and PLHIV)	For diagnosis: mWRD test, one outpatient visit with one contact with a healthcare provider for diagnosis of TB
Systematic screening of household contacts (aged < 15 years)	For screening: symptom screening + one community screening visit For diagnosis: mWRD test, one outpatient visit
Systematic screening of household contacts (aged ≥ 15 years)	For screening: symptom screening, one community screening visit, digital chest radiography (at health facilities) + CAD For diagnosis: mWRD test, one outpatient visit
TB infection testing of household contacts aged ≥ 5 years	IGRA test, one outpatient visit
Systematic screening of newly diagnosed PLHIV for TB or those who have been on ART for < 6 months (aged ≥ 10 years)	For screening: W4SS screening + C-Reactive protein test For diagnosis: mWRD test, one outpatient visit
Systematic screening of newly diagnosed PLHIV or those who have been on ART for < 6 months (aged < 10 years)	For screening: symptom screening For diagnosis: mWRD test, one outpatient visit
Systematic screening of PLHIV who have already been on ART for ≥ 6 months (aged ≥ 10 years)	For screening: W4SS screening + digital chest radiography + CAD * (at health facilities) For diagnosis: mWRD test, one outpatient visit
Systematic screening of PLHIV who have already been ART for ≥ 6 months (aged < 10 years)	For screening: symptom screening For diagnosis: mWRD test, one outpatient visit
High-risk groups in the community (aged ≥ 15 years): we assume that ~8% of the population in every high-TB burden country is at high risk for TB disease, with a TB incidence >2.5 times that in the general population.	For screening: symptom screening + digital chest radiography + CAD * (at health facilities) For diagnosis: mWRD test, one outpatient visit
TB preventive treatment to household contacts (age < 5 years)	3 months of Isoniazid and Rifampicin regimen, one outpatient visit
TB preventive treatment to household contacts (age ≥ 5 years)	3 months of Isoniazid and Rifapentine regimen, one outpatient visit

PLHIV = people living with HIV; ART = antiretroviral therapy; W4SS = WHO-recommended four-symptom screen; mWRD = molecular WHO-recommended rapid diagnostic tests (e.g., Xpert MTB/Rif^®^ test); * CAD = computer-aided detection software that uses artificial intelligence to analyze chest X-rays for the presence of abnormalities suggestive of pulmonary TB.

**Table 4:** Assumptions made to estimate the size of the target population requiring interventions and services to meet 2022 UNHLM targets for case detection and prevention.

Interventions	Target Population Size Estimated and Included in the Costing Model	Population Needing Intervention
Passive case finding	Adults (aged 15 years and above), children (aged < 15 years), pulmonary/extrapulmonary, HIV+/HIV−	10 persons tested for every person with TB detected
Systematic screening of household contacts of all people with TB diagnosed through passive case finding	Household contacts aged 0 to 5 years, 5–14 years, and >14 years.	100% of the estimate of the number of household contacts
Systematic screening of people living with HIV	Newly diagnosed people living with HIV (or those who have been on ART for <6 months) and those have already been on ART for > 6 months	100% of the estimate of the people living with HIV
High-risk groups in the community (8% of the country’s population with an estimated TB incidence 2.5 times that of the general population, or a TB prevalence ≥0.5%)	Age ≥ 15 years	80% of the high-risk group size
TB Preventive treatment to household contacts of bacteriologically confirmed TB disease	Age < 5 years, ≥5 years	90% of the estimated household contacts (of these, 20% will receive TPT after undergoing a TB infection test, and the remaining 80% will receive TPT directly without a TB infection test)

**Table 5:** Volumes of certain key interventions and weighted average unit costs across 33 high-burden countries required to achieve UNHLM TB case finding and TPT targets.

Service Component	Intervention/Activity	Volumes (2022)	Weighted Average Unit Cost in USD (2022)
Passive case finding	Xpert MTB/Rif test	45,089,952	21.94
Systematic screening of household contacts and TPT provision	Symptom screening	19,408,492	3.59
	CXR + CAD (for age ≥ 15 years)	12,679,959	10.71
	Xpert MTB/Rif test	4,718,458	21.53
	IGRA tests	3,129,988	16.44
	3 HR regimen	2,902,303	15.98
	3 HP regimen	15,649,941	18.52
Systematic screening of PLHIV	W4SS screening	23,122,906	5.40
	CRP	488,603	4.13
	CXR	22,325,116	6.06
	Xpert MTB/Rif test	7,275,816	22.42
Systematic screening of high-risk groups	Symptom screening	303,352,660	3.21
	CXR + CAD	288,185,027	9.95
	Xpert MTB/Rif test	30,335,266	21.36

**Table 6:** Numbers of people with TB to be detected with different approaches and TB preventive treatments to be provided in order to achieve the UNHLM 2018 targets in 33 high-TB burden countries, with estimates of the direct costs of different components.

	Item	Value
Numbers	Total number of people with TB to be detected	**12,617,976**
	Number to be detected through passive case finding (including systematic screening of people with HIV)	9,655,014
	Number to be detected through systematic screening of household contacts	779,736
	Number to be detected though systematic screening of high-risk group	2,183,226
	Number of household contacts of people with bacteriologically confirmed TB to be placed on TPT	**18,552,243**
Costs	Total direct costs	**6,710,981,010**
	Cost for passive case finding	991,489,828
	Cost for systematic screening of household contacts	287,902,333
	Cost for systematic screening of people with HIV (ART)	648,263,272
	Cost for systematic screening of high-risk groups (urban informal settings)	4,488,585,104
	Cost for TPT among household contacts	392,224,556

## Data Availability

The data presented in this study are available in the Supplementary Materials.

## Supplementary Materials

The following supporting information can be downloaded at https://www.mdpi.com/article/10.3390/tropicalmed8040214/s1. Supplementary Figures indicate the TB screening algorithms that were used for developing the costing model. Supplementary Table S1 shows the demographic and TB burden profile of the 33 countries included in this costing model.
