# Supplementary material for: Scaling Up TB Screening and TB Preventive Treatment Globally: Key Actions and Healthcare Service Costs
Source: Trop Med Infect Dis. 2023 Apr 1;8(4):214. doi: 10.3390/tropicalmed8040214 (PMC10144108; doi:10.3390/tropicalmed8040214)
Supplement: Supplementary file 1 [file tropicalmed-08-00214-s001.zip › 5.1_Supplementary_file_S1_algorithms_Services_costing.pptx]

## Slide 1
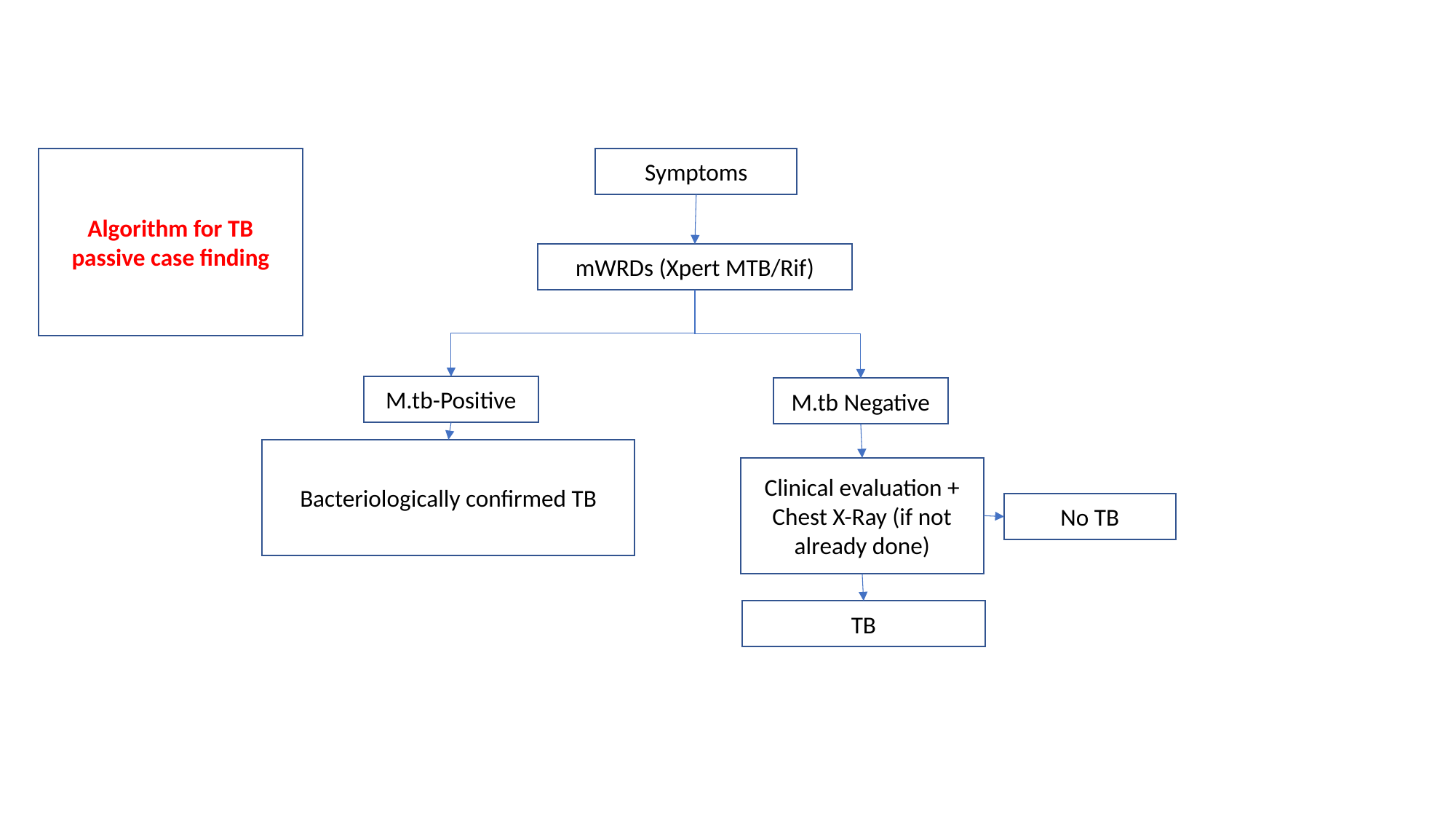

Algorithm for TB
passive case finding
Symptoms
mWRDs (Xpert MTB/Rif)
M.tb-Positive
M.tb Negative
Bacteriologically confirmed TB
Clinical evaluation + Chest X-Ray (if not already done)
No TB
TB

## Slide 2
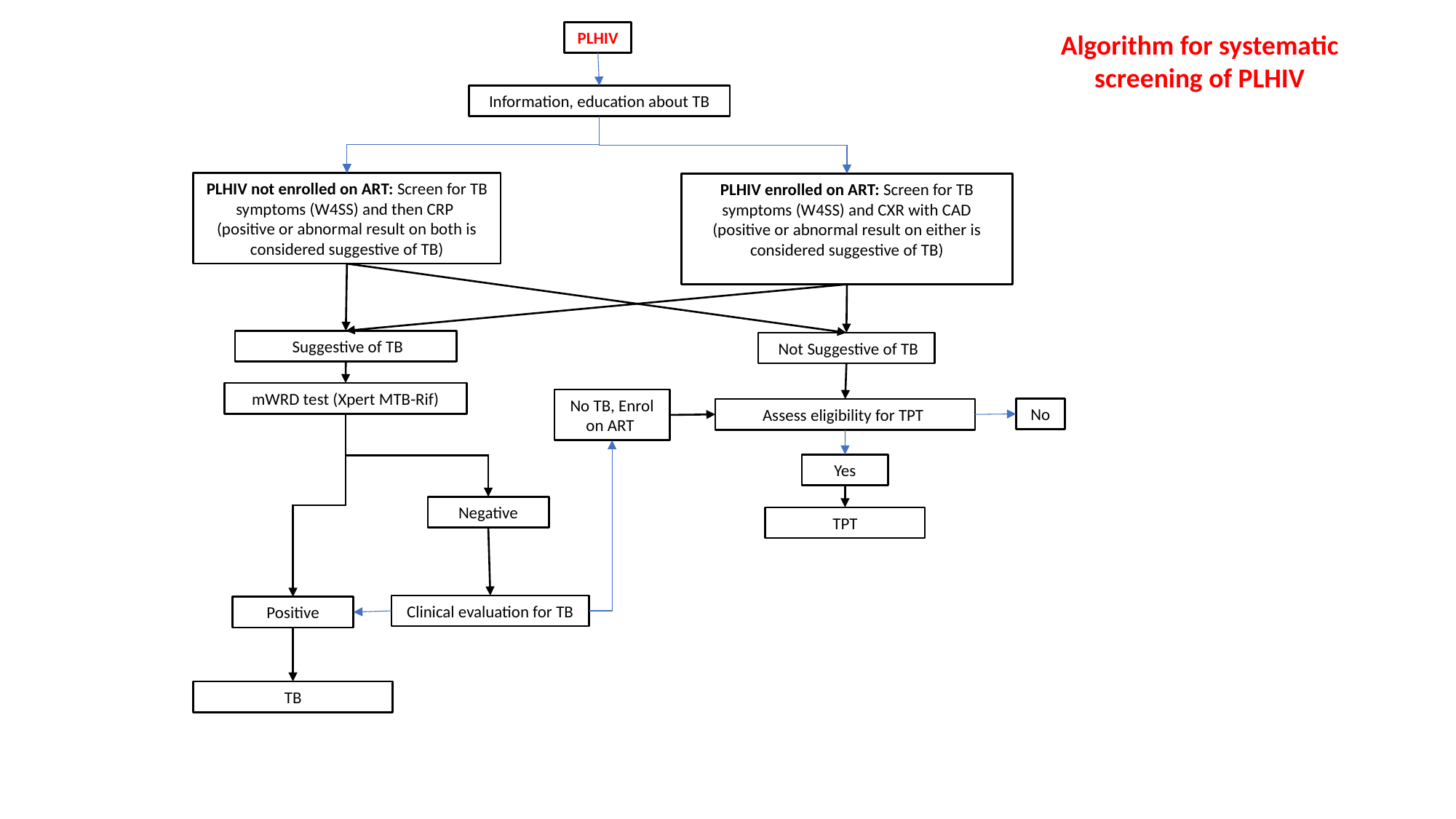

PLHIV
Information, education about TB
PLHIV not enrolled on ART: Screen for TB symptoms (W4SS) and then CRP
(positive or abnormal result on both is considered suggestive of TB)
PLHIV enrolled on ART: Screen for TB symptoms (W4SS) and CXR with CAD
(positive or abnormal result on either is considered suggestive of TB)
 Suggestive of TB
 Not Suggestive of TB
mWRD test (Xpert MTB-Rif)
No TB, Enrol on ART
No
Assess eligibility for TPT
Yes
Negative
TPT
Clinical evaluation for TB
Positive
TB
Algorithm for systematic screening of PLHIV

## Slide 3
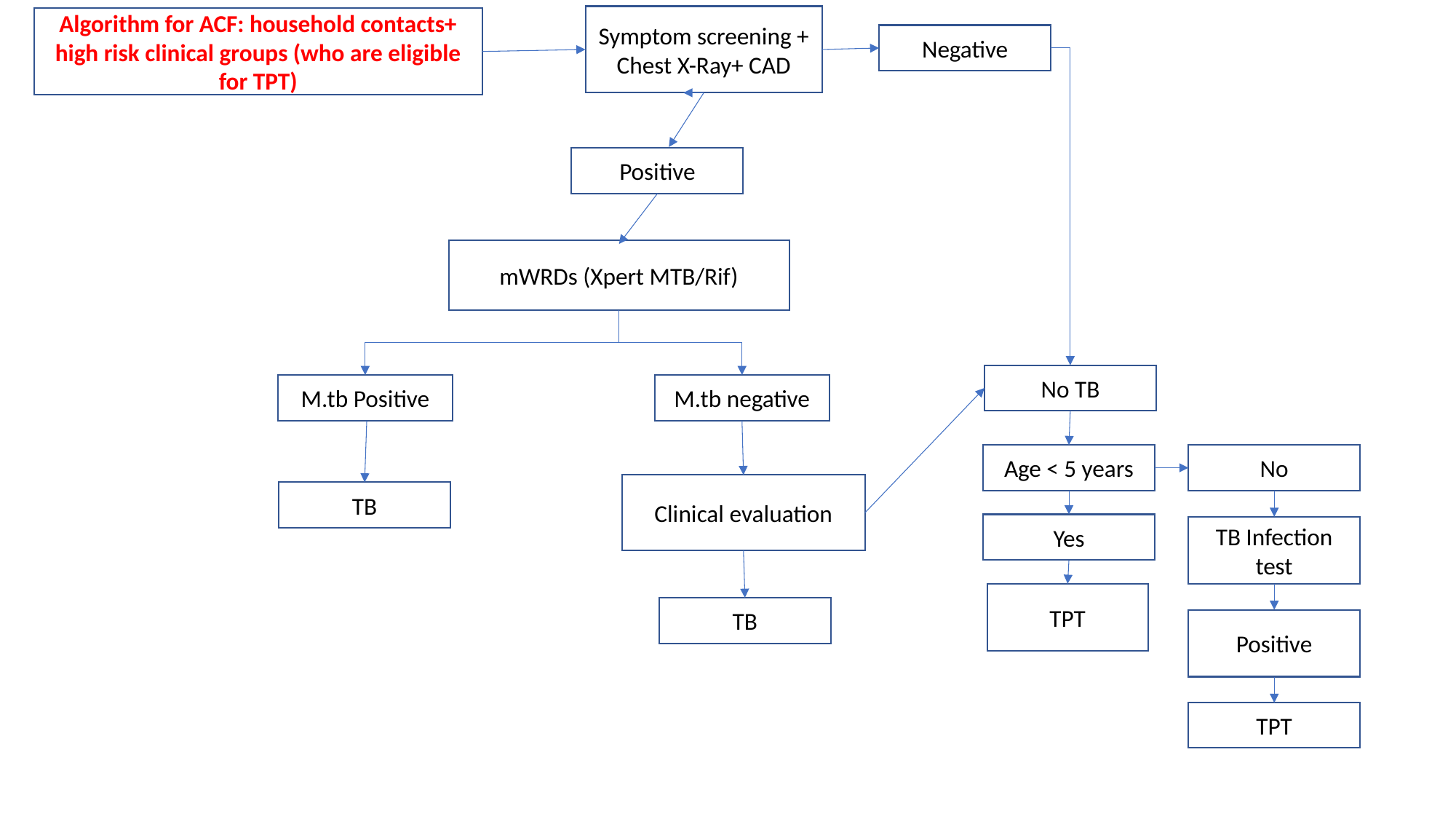

Symptom screening + Chest X-Ray+ CAD
Algorithm for ACF: household contacts+ high risk clinical groups (who are eligible for TPT)
Negative
Positive
mWRDs (Xpert MTB/Rif)
No TB
M.tb Positive
M.tb negative
Age < 5 years
No
Clinical evaluation
TB
Yes
TB Infection test
TPT
TB
Positive
TPT

## Slide 4
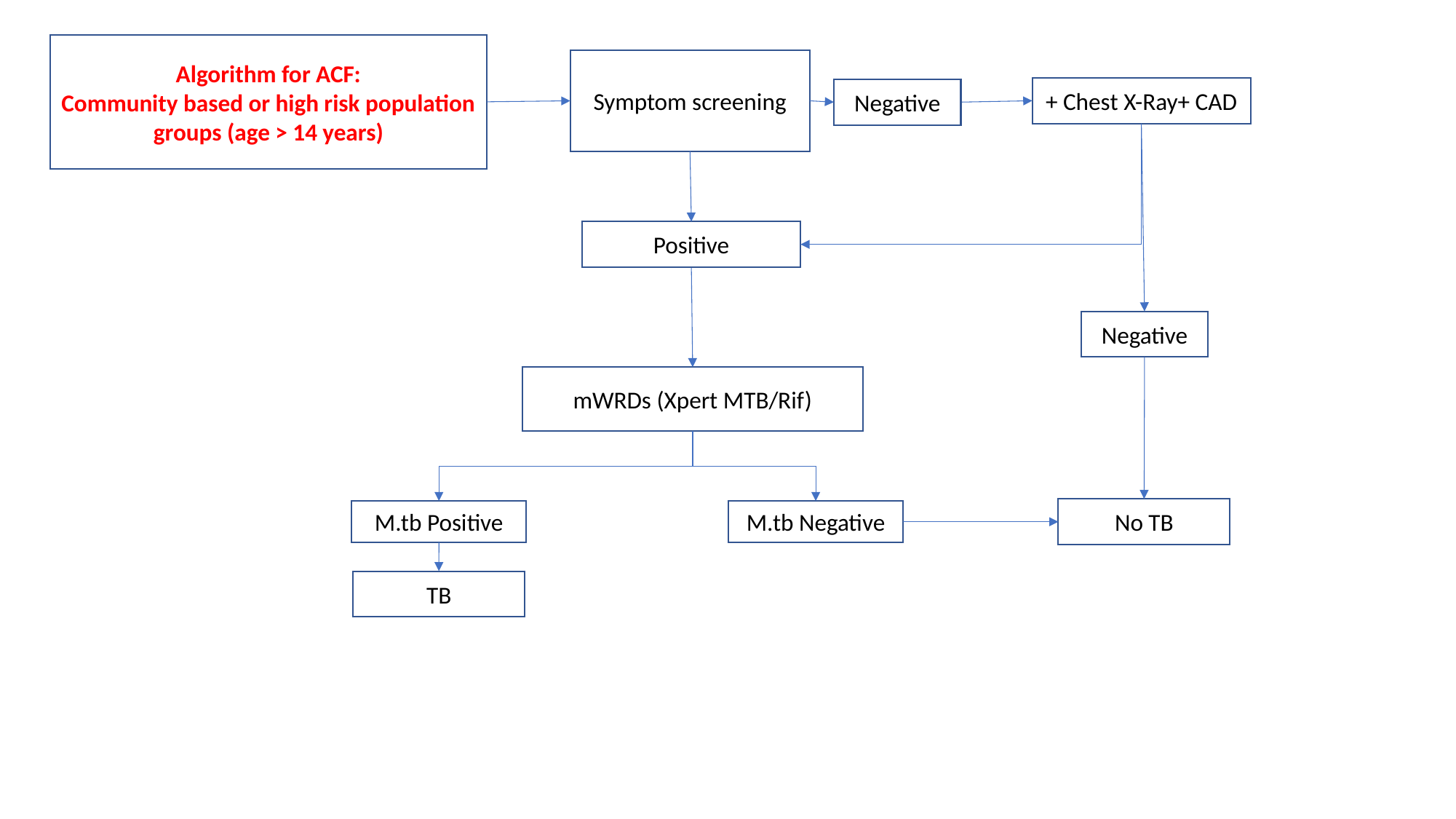

Algorithm for ACF:
Community based or high risk population groups (age > 14 years)
Symptom screening
+ Chest X-Ray+ CAD
Negative
Positive
Negative
mWRDs (Xpert MTB/Rif)
No TB
M.tb Positive
M.tb Negative
TB
